# Comparison of non-invasive ventilation use and outcomes in children with Down syndrome and other children using this technology

**DOI:** 10.3389/frsle.2023.1169236

**Published:** 2023-05-19

**Authors:** Rafiaa Valji, Maria L. Castro-Codesal, Melanie Lewis, Joanna E. MacLean

**Affiliations:** ^1^Divisions of Respiratory Medicine, Department of Pediatrics, Faculty of Medicine and Dentistry, University of Alberta, Edmonton, AB, Canada; ^2^Division of General and Community Pediatrics, Department of Pediatrics, Faculty of Medicine and Dentistry, University of Alberta, Edmonton, AB, Canada; ^3^Stollery Children's Hospital, Edmonton, AB, Canada; ^4^Women's and Children's Health Research Institute, Faculty of Medicine and Dentistry, University of Alberta, Edmonton, AB, Canada

**Keywords:** pediatric, obstructive sleep apnea, trisomy 21, continuous positive airway pressure, bilevel positive airway pressure

## Abstract

**Rationale:**

Children with Down syndrome (DS) make up a substantial portion of long-term non-invasive ventilation (LT-NIV) users though it is unclear if their unique features alter LT-NIV efficacy or use. The aim of this study is to compare the use and outcomes of LT-NIV for children with DS and a matched comparison (MCG).

**Methods:**

This is a sub-study of a 10-year retrospective review of children initiated on LT-NIV in Alberta, Canada (*N* = 622). Children with DS (*n* = 106) were matched in a 1:2 ratio with other children using LT-NIV based on age and therapy start date. Data was collected from medical and sleep laboratory records.

**Results:**

Upper airway disease was the most common indication for LT-NIV in both groups, though was higher in children with DS (DS: 90% vs. MCG: 50%, OR 8.64 [95% CI 4.38–17.04]). Sleep and respiratory parameters, at the baseline diagnostic sleep study and the change from baseline to treatment study, did not differ between groups. Nasal masks were the predominant mask type in both children with DS (55%) and the MCG (66%) with more children with DS, compared to the MCG, using full face masks (DS: 45 vs. MCG: 33%, *p* < 0.05). Continuous positive airway pressure was used more often in children with DS (93.3% vs. 69.2%, *p* < 0.001) while bilevel-positive airway pressure was more common in the MCG (DS: 6.7% vs. MCG 30.8%, *p* < 0.001). Children with DS were followed longer than children in the MCG (DS: 2.4 [IQR 2.8] vs. MCG: 1.8 [IQR 2.7] years, *p* < 0.05). Adherence was lower in children with DS at both 6–12 month follow-up and most recent visit with a similar decrease in adherence in both groups over the follow-up period (0.0 [IQR 1.4] vs. −0.3 [IQR 2.0]. Despite this, 66% and 49% of children with DS used LT-NIV for more than 4 h/night at the 6–12 month and most current visit, respectively. Discontinuation of LT-NIV and mortality did not differ between groups.

**Conclusion:**

LT-NIV is a common and efficacious treatment in children with DS used predominantly for upper airway obstruction. While adherence is lower, the majority of children with DS are successful at using LT-NIV.

## Introduction

Down syndrome (DS) is the most common chromosomal condition associated with intellectual disability occurring in ~1 in 800 births worldwide (Bull, 2020). Though there is phenotypic diversity, DS is characterized by multisystem involvement including otitis media, hearing loss, eye disease, congenital cardiac disease, hypothyroidism and respiratory disease including sleep-related breathing disorders (Bull et al., 2022). Obstructive sleep apnea (OSA) is reported to occur in 30–76% of children with DS as compared to 1.2–5.7% of neurotypical children; up to 50% of children with DS with OSA having moderate to severe disease (Hill et al., 2016; Maris et al., 2016; Lee et al., 2018; Nerfeldt and Sundelin, 2020; Waters et al., 2020; Bull et al., 2022). Craniofacial variations associated with DS, such as midface and mandibular hypoplasia, relative macroglossia, a narrow nasopharynx and short palate contribute to upper airway narrowing (Allareddy et al., 2016; Jayaratne et al., 2017; Takizawa et al., 2022). In addition, children with DS are at risk for airway stenosis, tracheobronchomalacia, and laryngomalacia as well as distal airway anomalies including lung hypoplasia, decreased airway branching, and reduced number of alveoli (Colvin and Yeager, 2017; Danopoulos et al., 2021). Upper and lower airway compromise is often compounded by muscle hypotonia, obesity, hypothyroidism, gastro-esophageal reflux, and aspiration (Lal et al., 2015). With both upper and lower airway compromise, children with DS are vulnerable to OSA as well as respiratory and cardiac disease that may result in respiratory impairment.

In addition to unique risk factors for OSA and respiratory impairment, children with DS have a poorer response to first line surgical treatment for OSA, most often adenotonsillectomy. While the definition of treatment failure differs between studies, 30–70% of children with DS require additional treatment after surgery (da Rocha et al., 2017; Dudoignon et al., 2017; Maris et al., 2017; Waters et al., 2020). For children with DS, up to 55% go on to use LT-NIV (including continuous positive airway pressure, CPAP, and bilevel positive airway pressure, BPAP) after incomplete resolution of OSA after surgery (Shete et al., 2010; Dudoignon et al., 2017). In a systematic review of LT-NIV use in children with DS, children with DS made up 18% of children using LT-NIV across 20 studies (Hudson et al., 2022). This is in keeping with a population study of LT-NIV use in children that reported 18% of children using LT-NIV were children with DS (Castro-Codesal et al., 2018). Despite this high incidence of use, our understanding of the use and outcomes of LT-NIV specific to children with DS is limited.

Given the inherent challenges for children with DS with respect to cognitive ability, behavioral concerns, obesity, and pulmonary hypertension that may be compounded by untreated OSA or sleep-related hypoventilation, understanding the balance of burden vs. benefit of LT-NIV use for children with DS is important. LT-NIV is an effective and well-tolerated therapy with potential benefits such that consideration of its use should not differ for children with and without DS (Hudson et al., 2022). What is not known is whether LT-NIV use and outcomes differ between children with and without DS such that a different approach to LT-NIV use is needed for children with DS. Accordingly, the aim of this study is to compare the use and outcomes of LT-NIV children with DS to a matched comparison group of children using LT-NIV.

## Materials and methods

This retrospective cross-sectional study is part of a larger study of 622 children started on long-term NIV in the province of Alberta from January 2005 to December 2014 (Castro-Codesal et al., 2018). Children (0–17 years of age) were included if they used NIV for a minimum of 3 months outside of an acute care setting. NIV was defined as breathing support provided through an interface outside the airway where breathing support including both continuous (i.e., single positive airway pressure) and bilevel (i.e., inspiratory and expiratory pressures) positive airway pressure. Children with DS were matched in a 1:2 ratio with children in the larger cohort based on age and initiation date within 6 months (i.e., ±6 months for both age and date of initiation) to form a matched comparison group (MCG). The Health Research Ethics Boards at both participating institutions approved the study protocol.

Data was abstracted from the medical charts and sleep laboratory records of all children using LT-NIV at the two children's hospitals in Alberta, Canada. Since these hospitals have the only publicly funded pediatric sleep laboratories in Alberta, they capture most children using LT-NIV in the province. Data were collected at baseline, 6–12 months after initiation, and the most recent clinic visit. Study data were collected and managed using REDCap electronic data capture tools hosted and supported by the Women & Children's Health research institute at the University of Alberta (Harris et al., 2009).

Data collection included demographics, primary disease condition leading to LT-NIV, co-morbidities, and history of previous upper airway surgery. The primary indications for LT-NIV were grouped into five broad categories: upper airway, central nervous system, musculoskeletal/neuromuscular, respiratory (excludes upper airway), cardiac, and other conditions. Data collected on NIV technology included location of NIV start, NIV type, primary mask type, adherence data from NIV machine downloads (average hours of use/night, % days >4 h use), and use of additional technology. Location of NIV start included home, hospital ward, or the neonatal/pediatric intensive care units; home was further divided into “pre-titration,” where children started NIV with pressure adjustments at home prior to a PSG treatment study, and “post-titration,” where a PSG treatment study preceded starting NIV at home. Outcomes included discontinuation of NIV, reasons for discontinuation (improvements in the underlying condition, patient/family decision to stop NIV, transfer of services, switch to invasive mechanical ventilation, or other), and mortality.

Polysomnography (PSG), including diagnostic, treatment, and split night (including a separate diagnostic and titration portions in the same night) studies, were performed using standard laboratory procedures by a sleep technologist. Studies collected before 2007 were sleep staged according to Anders et al. (1971) (<6 months of age) or Rechtschaffen (1968) (≥6 months of age) with scoring of respiratory variables based on the American Thoracic Society and previously published data in children (Marcus et al., 1992; American Thoracic Society, 1996). The guidelines of the American Academy of Sleep Medicine (Iber et al., 2007) and subsequent updates were applied to scoring starting in 2007. Apnea-hypopnea index (AHI) was calculated using the number of apneas and hypopneas that occurred during sleep divided by the total sleep time (TST). Obstructive AHI (OAHI) was defined as the sum of the obstructive and mixed apneas and obstructive and mixed hypopneas divided by the TST. A minimum TST of 120 min was required for PSGs to be included in the analysis; this cut-off was chosen as studies with TST <120 min are deemed “insufficient data” and considered for a repeat study in this laboratory. For analysis, the diagnostic portion of split night and full night studies were combined as were the treatment portions of split night and full night studies.

Chi-square test or Fisher's exact test, and Mann-Whitney U test were used to compare categorical and continuous variables between groups, respectively. Wilcoxon Signed Ranks test was used to compare sleep and respiratory parameters between paired diagnostic and titration PSG studies. Odds ratios or Mann-Whitney U test statistic were calculated to determine a measure of association for two dichotomous or continuous variables, respectively. Multi-variable analysis of the outcomes of adherence and discontinuation of LT-NIV were performed using linear or binary logistic regression as appropriate. Down syndrome was included in all models. Additional variables were selected from univariable analysis (Tables 1–**3**) and tested step-wise. Variables were kept in the multi-variable model if the *p*-value for the corresponding coefficient was <0.05. Excluded variables were then tested to determine if they improved the model with the final model based on significant improvement to the model based on the highest adjusted *R*^2^ or −2 log likelihood as appropriate. A *p*-value of <0.05 was used to support statistically significant effects. Data analyses were performed using IBM SPSS Statistics version 24.0 (SPSS, Inc., Chicago, IL).

**Table 1:** Comparison of baseline clinical characteristics, including respiratory parameters, for children with Down syndrome and a matched comparison group using long-term non-invasive ventilation.

**Characteristic**	**Down syndrome (*n* = 106)**	**Matched comparison group (*n* = 212)**	**Odds ratio (OR 95% CI) or Mann-Whitney U test statistic**
Age (y) at NIV start date, median (IQR)^**^	5.8 (10.4)	6.0 (10.2)	−0.09 (−1.34 to 1.16)
Males (*n*, %)	64 (60.4)	125 (59.0)	11128
**Underlying disease category (** ***n*** **, %)**			
Upper airway	95 (89.6)	106 (50.0)	8.64 (4.38 to 17.04)^‡^
Central nervous system	0 (0.0)	51 (24.1)	NA
Musculoskeletal/neuromuscular	3 (2.8)	32 (15.1)	0.16 (0.05 to 0.55)^‡^
Respiratory	3 (2.8)	14 (6.6)	0.41 (0.12 to 1.47)
Cardiac	5 (4.7)	2 (0.9)	5.20 (0.99 to 27.25)^*^
Other	0 (0.0)	7 (3.3)	1.03 (1.01 to 1.06)
**Surgical status**			
Prior A/T (*n*, %)	60 (58.3)	93 (45.4)	1.68 (1.04 to 2.71)^*^
No A/T	43 (41.7)	112 (54.6)	
A/T surgical status unknown	3	7	
**Baseline diagnostic PSG data, median (IQR)**			
TST (min)	309.4 (184.9)	296.5 (226.5)	9440.00
Sleep efficiency (%)	85.1 (14.1)	84.9 (19.0)	9175.00
REM sleep (%)	14.1 (10.6)	16.3 (11.6)	7344.00^*^
Arousal index (events/h)	8.2 (10.2)	8.9 (11.6)	8462.00
Total AHI (events/h)	12.1 (20.2)	12.0 (25.5)	9420.00
Obstructive AHI (events/h)	11.1 (14.3)	8.8 (22.9)	7954.50
Central AHI (events/h)	0.3 (3.2)	1.2 (3.6)	7664.00
% TST with SpO_2_ <90%	0.9 (10.1)	0.5 (6.3)	8446.50
% TST with TcCO_2_ >50 mmHg	4.7 (36.2)	0.35 (29.3)	6270.00

^**^Mean difference from independent samples *t*-test.

AHI, apnea-hyponea index; A/T, adenoidectomy, tonsillectomy or both; CI, confidence interval; h, hour; IQR, intra-quartile range; min, minute; REM, rapid eye movement sleep; SpO2, pulse oxygen saturation; TcCO2, transcutaneous carbon dioxide; TST, total sleep time; y, year.

^*^p < 0.05;^‡^*p* < 0.001.

## Results

A total of 121 children with DS were identified in the data set, 106 of whom could be matched to 212 non-DS children for inclusion in this study. Age and sex distribution did not differ between groups (Table 1). The most common reason for LT-NIV use was upper airway indications in both groups though the proportion was higher in the DS compared to the MCG group. Cardiac indications were more common in children with DS while central nervous system and musculoskeletal/neuromuscular indications were more common in non-DS children. More children with DS had undergone adenoidectomy, tonsillectomy or both compared to the MCG. Rates of split night studies were similar between groups for the initial diagnostic PSG (DS 55% vs. MCG 50%) and titration PSG (DS 47% vs. MCG 39%). Baseline sleep and respiratory parameters differed only for percentage rapid eye movement (REM) sleep where children with DS had a lower percentage of REM sleep compared to the MCG.

There were few differences with respect to NIV technology between groups (Table 2). The location of LT-NIV initiation and use of additional technologies did not differ between groups. Nasal masks were the most common mask for both groups though a higher proportion of children with DS used full face masks compared to the MCG. CPAP was the most common NIV technology in both groups though CPAP use was higher and BPAP lower in children with DS compared to the MCG.

**Table 2:** Comparison of technology use for children with Down syndrome and a matched comparison group using long-term non-invasive ventilation.

**NIV technology**	**Down syndrome (*n* = 106)**	**Matched comparison group (*n* = 212)**	**Odds ratio (OR 95% CI) or Mann-Whitney U test statistic**
**Location of NIV start (** ***n*** **, %)**			
Home (pre-titration)	29 (27.4)	51 (24.1)	1.19 (0.70 to 2.02)
Home (post-titration)	59 (55.7)	108 (50.9)	1.21 (0.76 to 1.93)
Hospital ward	10 (9.4)	24 (11.3)	0.82 (0.38 to 1.78)
NICU/PICU	8 (7.5)	29 (13.7)	0.52 (0.23 to 1.17)
**Interface type (** ***n*** **, %) at start ventilation visit**			
Nasal	53 (54.6)	126 (66.3)	0.61 (0.37 to 1.00)
Full face	44 (45.4)	62 (32.6)	1.71 (1.04 to 2.83)^*^
Nasal pillow	0 (0)	2 (1.1)	1.01 (1.00 to 1.03)
Interface type unknown	9	22	
**NIV type (** ***n*** **, %) at start ventilation visit**			
CPAP^**^	98 (93.3)	146 (69.2)	6.23 (2.74 to 14.16)^‡^
BPAP	7 (6.7)	65 (30.8)	0.16 (0.07 to 0.36)^‡^
NIV type unknown	1	1	
Use of one or more additional technology (at baseline visit) (*n*, %)	10 (9.4)	17 (8.0)	1.20 (0.53 to 2.71)
Nasogastric or gastrostomy feeding tube	9 (8.5)	17 (8.0)	1.06 (0.46 to 2.48)
Oxygen	2 (1.9)	1 (0.5)	4.06 (0.36 to 45.27)

^**^Includes auto-PAP for 1 child with DS and 2 non-DS children.

BPAP, bilevel positive airway pressure; CPAP, continuous positive airway pressure; NICU, neonatal intensive care unit; NIV, non-invasive ventilation; PAP, positive airway pressure; PICU, pediatric intensive care unit.

^*^p < 0.05;^‡^*p* < 0.001.

The period of follow-up was longer in children with DS compared to the MCG with a mean difference of 5½ months (Table 3). Treatment response, as measured by change in PSG parameters from baseline to first treatment study, did not differ between groups. Nor did LT-NIV discontinuation, reasons for discontinuation, or mortality with low mortality in both groups. Adherence data was available for 150 children (47%; 47 children with DS, 103 non-DS children). Adherence, both average hours of use/night and % nights with >4 h of use, was lower in children with DS compared to the MCG at both follow-up visits though the change in adherence between these visits did not differ between groups. Despite lower adherence, 66% and 49% of children with DS used LT-NIV for more than 4 h/night at the 6–12 month follow-up and most current visit, respectively.

**Table 3:** Comparison of treatment response, adherence and discontinuation for children with Down syndrome and a matched comparison group using long-term non-invasive ventilation.

**Treatment response and outcomes**	**Down syndrome (*n* = 106)**	**Matched comparison group (*n* = 212)**	**Odds ratio (OR 95% CI) or Mann-Whitney U test statistic**
Follow up period (y), median (IQR)	2.4 (2.8)	1.8 (2.7)	8782.50^*^
PSG indices, median differences^§^ (IQR):	Change from Dx to Tx	Change from Dx to Tx	
TST (min)	16.6 (221.4)	−17.5 (271.6)	5071.50
Sleep efficiency (%)	1.8 (18.7)	1.3 (19.9)	5085.00
REM sleep (%)	2.9 (15.0)	1.9 (16.3)	4245.00
Arousal index (events/h)	−1.5 (9.3)	−1.7 (7.9)	4382.00
Obstructive AHI (events/h)	−5.0 (13.6)	−3.8 (14.4)	4334.50
Central AHI (events/h)	0.0 (1.6)	0.0 (3.6)	4259.50
% TST with SpO2 <90%	−0.5 (8.9)	−0.2 (3.4)	4813.00
% TST with TcCO2 > 50 mmHg	−0.2 (24.7)	0.0 (13.7)	3381.50
Adherence (median, IQR):^***^			
Average use/night (h), 6-12 mos visit	5.2 (4.2)	7.6 (3.6)	1160.00^‡^
Average use/night (h), most recent visit	4.6 (5.7)	8.0 (3.4)	1189.50^‡^
Change in use/night (h)	0.0 (1.4)	−0.3 (2.0)	758.00
% days with use >4 h, 6–12 mos visit	54.3 (66.3)	84.6 (46.5)	1577.00^‡^
% days with use>4 h, most recent visit	38.0 (48.8)	88.0 (48.5)	1436.00^‡^
Change in % days with use>4 h	−2.3 (20.8)	0.0 (18.4)	758.00
Discontinuation of NIV^**^ (*n*, %)	56 (56.0)	124 (59.3)	0.87 (0.54 to 1.41)
Reasons for discontinuation (*n*, %):			
Improvement in underlying condition	12 (21.4)	39 (31.5)	0.59 (0.28 to 1.25)
Child/family decision to stop NIV	20 (35.7)	30 (24.2)	1.74 (0.88 to 3.45)
Transfer of services	15 (26.8)	27 (21.8)	1.31 (0.63 to 2.73)
Switch to IMV	0 (0.0)	4 (3.2)	NA
Other	9 (16.0)	24 (19.4)	1.25 (0.54 to 2.91)
Mortality (*n*, %)	3 (2.8)	9 (4.2)	0.66 (0.17 to 2.48)

^§^From baseline DX PSG to first Tx PSG.

^**^Excludes mortality as a reason for discontinuation. *n* = 9 lost to follow-up.

^***^Adherence data available from 150 children (47%; 47 children with DS, 103 non-DS children).

AHI, apnea-hypopnea index; Dx, diagnostic; h, hour; IQR, intra-quartile range; NIV, non-invasive ventilation; min, minute; PSG, polysomnography; REM, rapid eye movement; SpO_2_, pulse oxygen saturation; TcCO_2_, transcutaneous carbon dioxide; TST, total sleep time; Tx, treatment; y, year.

^*^p < 0.05;^‡^*p* < 0.001.

The multi-variable analyses of adherence show that DS and CPAP are predictors of lower adherence while longer duration of follow-up is a predictor of higher adherence (Table 4). The *R*^2^ is low for all models demonstrating that these models explain little of the overall variance in adherence. For the outcome of NIV discontinuation (Table 5), children using CPAP and of female sex are more likely to discontinue LT-NIV while having DS is not an overall predictor. Variables predicting discontinuation because of improvement in the underlying condition include use of CPAP and younger age while children with DS are less likely to discontinue because of improvements in the underlying condition. Predictors of discontinuation because of child/family decision to stop NIV include having DS and longer duration of follow-up.

**Table 4:** Results of multivariable analysis with adherence at 6–12 month and most recent visit as outcomes variables, and clinical characteristics and technology use as predictors in children with Down syndrome and a matched comparison group.

**Variables**	**Co-variates**	**B ±SE**	**Beta**	**95% CI for B**
Percent nights >4 h of use at 6-12 mos follow up visit^a^	Down syndrome	−15.70 ± 5.66^†^	−0.218	−26.88 to −4.52
	CPAP	−24.35 ± 6.64^‡^	−0.288	−37.47 to −11.22
	Constant	88.17 ± 5.77^‡^		76.78 to 99.57
Percent nights >4 hours of use at most recent visit^b^	Down syndrome	−21.10 ± 5.97^‡^	−0.288	−32.90 to −9.31
	Constant	69.13 ± 3.49^‡^		62.22 to 76.04
Average h of use/night at 6-12 mos follow-up visit^c^	Down syndrome	−1.98 ± 0.57^‡^	−0.274	−3.10 to −0.86
	CPAP	−2.68 ± 0.67^‡^	−0.315	−4.01 to −1.36
	Constant	9.37 ± 0.58		8.22 to 10.52
Average h of use/night at most recent visit^d^	Down syndrome	−2.30 ± 0.55^‡^	−0.339	−3.396 to −1.20
	Follow up duration	0.29 ± 0.11^*^	0.215	0.072 to 0.52
	Constant	6.76 ± 0.45		5.89 to 7.66

A/T, adenoidectomy, tonsillectomy or both; CPAP, continuous positive airway pressure; mos, month.

^*^p < 0.05;^†^*p* < 0.01;^‡^*p* < 0.001.

Model adjusted *R*^2^ ± SE: ^a^0.151 ± 30.98; ^b^0.076 ± 33.51; ^c^0.21 ± 3.01; ^d^0.141 ± 3.00.

**Table 5:** Results of multivariable analysis with discontinuation of non-invasive ventilation and reasons for discontinuation as outcome variables, and clinical characteristics and technology use as predictors in children with Down syndrome and a matched comparison group.

**Variables**	**Co-variates**	**B ±SE**	**Exp(B)**	**95% C.I. for Exp(B)**
NIV discontinuation^a^	Down syndrome	−0.42 ± 0.27	0.66	0.39 to 1.11
	CPAP	1.03 ± 0.29^‡^	2.80	1.57 to 4.98
	Sex (Female)	0.68 ± 0.25^†^	1.97	1.22 to 3.21
	Constant	−0.71 ± 0.26^†^	0.49	
Discontinuation reason: improvement in underlying condition^b^	Down syndrome	−0.82 ± 0.41^*^	0.44	0.20 to 0.98
	CPAP	1.70 ± 0.61^†^	5.46	1.65 to 18.06
	Age (y)	0.12 ± 0.36^‡^	1.13	1.06 to 1.21
	Constant	0.48 ± 0.38	1.62	
Discontinuation reason: child/family decision to stop NIV^c^	Down syndrome	0.74 ± 0.37^*^	2.10	1.03 to 4.31
	Follow-up duration (y)	0.33 ± 0.12^†^	1.39	1.09 to 1.77
	Constant	−0.20 ± 0.10	0.81	

A/T, adenoidectomy, tonsillectomy or both; CNS, central nervous system; CPAP, continuous positive airway pressure; MSK/NMD, musculoskeletal/neuromuscular disease; y, years.

^*^p < 0.05;^†^*p* < 0.01;^‡^*p* < 0.001.

-2 log likelihood: ^a^397.2, ^b^191.9; ^c^201.8.

## Discussion

The study results highlight that children with DS using LT-NIV demonstrate some unique features, though exhibit more similarities, when compared to other children using LT-NIV. The predominant reason for LT-NIV use in both groups is upper airway disease though children with DS had a higher rate of upper airway and cardiac disease. Prior removal of adenoids and/or tonsils, and full-face mask use are also more common in children with DS. Both groups show similar improvements in sleep and breathing parameters on PSGs in response to NIV as well as similar use of additional technology, reasons for stopping LT-NIV, and mortality rates. DS was an independent predictor of lower adherence for all four adherence measures. While having DS was not an independent predictor of overall discontinuation, children with DS had a lower likelihood of discontinuation because of improvement in the underlying condition and higher likelihood of discontinuation because of child/family decision to cease LT-NIV use.

There is limited information on the outcomes of LT-NIV use for cardiac indications in children including in children with DS specifically. Cardiac indications for LT-NIV use in children are relatively uncommon with reported rates between 1.4% to 10% across studies including cardiac indications as a category (Nathan et al., 2017; Castro-Codesal et al., 2018; Waters et al., 2020). In the present study, children with DS had a higher rate of starting LT-NIV use for cardiac indications than other children. Given that approximately half of live born children with DS will have congenital heart disease (CHD) (Dimopoulos et al., 2023), 28% will have pulmonary hypertension (Bush et al., 2018), and 17% to 56% will have OSA and pulmonary hypertension (Rabes et al., 2022), cardiac indications for LT-NIV are uncommon though important. There is, however, little information on the effects of LT-NIV on cardiac pathophysiology in the context of CHD or pulmonary hypertension including in children with DS. CPAP used for the treatment of OSA in children with DS may improve left ventricular (LV) function (Konstantinopoulou et al., 2016). A recent meta-analysis reporting on the effects of CPAP on cardiac mechanics in adults with OSA showed that LV systolic function increased during CPAP, and LV mechanics improved after CPAP treatment while improvements in right ventricular (RV) function were not detectable with conventional measures (Tadic et al., 2022). Studies of the effect of CPAP on RV function in adults, where RV function is measured by right heart catheterization, have failed to show change in resting pulmonary arterial pressure (Adir et al., 2021). In the context of a right-to-left cardiac shunt, CPAP may increase, rather than decrease, shunting in some patients (Tanaka et al., 2020). Given the range of CHD in DS and the potential effects of LT-NIV on cardiac pathophysiology, consultation with a pediatric cardiologist should be strongly considered before initiation of LT-NIV in a child with DS for cardiac indications or a history of CHD.

Adenotonsillectomy is first line therapy for children with OSA and enlarged lymphoid tissue in the absence of surgical contraindications including in children with DS (Marcus et al., 2012). In a health-administrative study of sleep-related surgeries performed between 1997 and 2012, adenotonsillectomy was the most common procedure performed in both children with DS and non-syndromic children (Ong et al., 2018). A recent medical chart review of children with DS undergoing polysomnography reported that airway surgery was offered to 64% of children with DS and OSA; 53% of those who underwent surgery and had a post-operative PSG showing no or mild OSA (Waters et al., 2020). In a study of children with DS who were initiated on CPAP for OSA, 53% of children had prior adenotonsillectomy or adenoidectomy alone (O'Donnell et al., 2006). In the current study, a higher proportion of children with DS, compared to the MCG, underwent adenoidectomy, tonsillectomy or both prior to LT-NIV initiation compared to other children which likely reflects the higher rate of upper airway indications for LT-NIV use in children with DS. Despite a lower OSA cure rate after upper airway surgery for OSA, surgery remains the first line treatment of OSA in children with DS with LT-NIV reserved for those with residual OSA needing treatment after surgery.

Adherence to LT-NIV remains a challenge for both children and adults using this treatment including in children with DS. Adherence is a complex issue with multiple contributors including health-related factors, psychosocial factors, and issues related to interaction with NIV technology (Pascoe et al., 2019; Perriol et al., 2019; Bhattacharjee et al., 2020; Katz et al., 2020). Previous studies comparing LT-NIV use in children with DS to other children are limited. In a study of children with and without DS using LT-NIV, children with DS showed a higher proportion of days used though similar proportion of days with use >4 h (MacDonagh et al., 2021). This was despite higher leak rates on machine downloads. Additional studies reported no difference in adherence in children with and without impaired cognition as well as higher rates of never establishing LT-NIV use in children with DS (O'Donnell et al., 2006; Amaddeo et al., 2018). Studies exclusively on children with DS support the potential for high adherence as well as adherence rates that are typical of children using LT-NIV (Hudson et al., 2022). The current study shows adherence rates are lower in children with DS though not out of keeping with rates reported in other cohorts of children using LT-NIV. DS is an independent predictor of lower adherence and a child/family decision to stop LT-NIV though these factors explain little of the variance in adherence or stopping LT-NIV. Investing in children with DS getting on track early may be beneficial for closing potential gaps between children with DS and other children with respect to adherence and continued LT-NIV use.

There is little data on the outcomes of LT-NIV use in children including for children with DS specifically. LT-NIV use in children with neuromuscular disease shows benefit for mortality, hospitalization, and PSG parameters for some sub-groups (AlBalawi et al., 2022). For infants, LT-NIV use improves respiratory sleep parameters for upper airway conditions and reduces hospitalizations as well as improves survival for spinal muscular atrophy type-1 (Bedi et al., 2018). In children with severe, complex neurological disabilities, LT-NIV improves respiratory sleep parameters though with no clear benefit for hospitalizations (Morrison et al., 2022). Mortality varies across studies of LT-NIV with estimates between 3.5% and 17% (MacLean and Fauroux, 2023). Outcomes data in children with DS as a group is limited though suggests favorable impact on attention and cardiac function (Hudson et al., 2022). The current study shows that, compared to other children, children with DS using LT-NIV show similar improvements in sleep and breathing parameters on PSGs, overall discontinuation rates, and mortality. Further work is needed to understand the impact of successful LT-NIV use in children with DS on broader health outcomes including cognition and quality of life.

Limitations to this study must be acknowledged. The study included only children who were successful at using NIV for at least 3 months which excluded children who were unsuccessful at initiating LT-NIV. Data collection was retrospective from chart review so relies on documentation by healthcare practitioners rather than direct measures from children using LT-NIV or their parents/caregivers. Subjective outcomes, such as reason for discontinuing LT-NIV, may be biased by healthcare practitioner impressions, and may be better measured by child/family report in future studies. Adherence data was available for just under ½ of the children with several potential contributing factors. The study period covered a time when fewer machines had internal modems. Alberta has a low population density with 18% of the population living outside metropolitan areas (Focus on Geography Series, 2022). Families needing to travel to provide SD cards for downloads and those with poor access to internet services in rural areas may have contributed to the low rate of available adherence data. Pressure adjustments during treatment studies mean that the data collected does not represent a steady state. That will, if anything, reduce the difference between the results of a diagnostic vs. treatment study such that our results may underestimate the treatment effect of LT-NIV. Children who initiated LT-NIV closer to the end of the data collection had less time to reach outcomes such that results may overestimate the proportion of children who successfully continue LT-NIV. The follow-up period, however, was longer for children with DS which means that children with DS, if anything, had more time to reach the outcome of LT-NIV discontinuation.

## Conclusion

Our results demonstrate that, while children with DS have some unique features, they share considerable similarities with other children using LT-NIV. Efficacy of LT-NIV, based on change in PSG results, does not differ between DS and other children using LT-NIV though adherence is lower in children with DS. Despite this, many children with DS persist with LT-NIV. Investment in understanding factors that may impact adherence early in the process of initiation and prospective studies of outcomes are needed to reduce the challenges and optimize LT-NIV use in children with DS.

## Data availability statement

The raw data supporting the conclusions of this article will be made available by the authors, without undue reservation.

## Ethics statement

The studies involving human participants were reviewed and approved by Human Research Ethics Committee, University of Alberta. Written informed consent from the participants' legal guardian/next of kin was not required to participate in this study in accordance with the national legislation and the institutional requirements.

## Author contributions

RV conceptualized the study, analyzed the data, prepared the first draft of the study, and critically reviewed and revised the manuscript. MC-C contributed to the data collection and critically reviewed and revised the manuscript. ML conceptualized the study and critically reviewed and revised the manuscript. JM conceptualized the study, analyzed the data, critically reviewed and revised the manuscript, and provided oversight for the study. All authors approved the final version of the manuscript for submission.

## References

[B1] Adir Y. Humbert M. Chaouat A. (2021). Sleep-related breathing disorders and pulmonary hypertension. Eur. Resp. J. 57, 2002258. 10.1183/13993003.02258-2020 32747397

[B2] AlBalawi M. M. Castro-Codesal M. Featherstone R. Sebastianski M. Vandermeer B. Alkhaledi B.. (2022). Outcomes of long-term noninvasive ventilation use in children with neuromuscular disease: systematic review and meta-analysis. Ann. Am. Thorac. Soc. 19, 109–119. 10.1513/AnnalsATS.202009-1089OC 34181865

[B3] Allareddy V. Ching N. Macklin E. A. Voelz L. Weintraub G. Davidson E.. (2016). Craniofacial features as assessed by lateral cephalometric measurements in children with Down syndrome. Prog. Orthod. 17, 35. 10.1186/s40510-016-0148-7 27722998 PMC5097953

[B4] Amaddeo A. F. Touil A. Khirani S. Griffon S. Fauroux L. Outpatient initiation B.. (2018). of long-term continuous positive airway pressure in children. Pedia. Pulmonol. 53, 1422–1428. 10.1002/ppul.24138 30070059

[B5] American Thoracic Society (1996). Standards and indications for cardiopulmonary sleep studies in children. American thoracic society. Am. J. Respirat. Crit. Care Med. 153, 866–878. 10.1164/ajrccm.153.2.8564147 8564147

[B6] Anders T. R. Emede R. Parmalee A. (1971). A Manual of Standarized Terminology: Techniques and Criteria for Scoring States of Sleep and Wakefulness in Newborn Infants. Los Angeles: Brain Information Service/Brain Research Institute (1971).

[B7] Bedi P. K. Castro-Codesal M. L. Featherstone R. AlBalawi M. M. Alkhaledi B. Kozyrskyj A. L.. (2018). Long-term non-invasive ventilation in infants: A systematic review and meta-analysis. Front. Pediatr. 6, 13. 10.3389/fped.2018.00013 29484287 PMC5816035

[B8] Bhattacharjee R. Benjafield A. V. Armitstead J. Cistulli P. A. Nunez C. M. Pepin J. D.. (2020). Adherence in children using positive airway pressure therapy: a big-data analysis. Lancet Digit. Health. 2, e94–e101. 10.1016/S2589-7500(19)30214-6 33334566 PMC7847310

[B9] Bull M. J. (2020). Down syndrome. N. Engl. J. Med. 382, 2344–2352. 10.1056/NEJMra1706537 32521135

[B10] Bull M. J. Trotter T. Santoro S. L. Christensen C. Grout R. W. GENETICS TCO. (2022). Health supervision for children and adolescents with Down syndrome. Pediatrics 149, 7010. 10.1542/peds.2022-057010 35490285

[B11] Bush D. Galambos C. Ivy D. D. Abman S. H. Wolter-Warmerdam K. Hickey F.. (2018). Clinical characteristics and risk factors for developing pulmonary hypertension in children with Down syndrome. J. Pediat. 202, 212–219.e212. 10.1016/j.jpeds.2018.06.031 30025669

[B12] Castro-Codesal K. Bedi P. K. Bendiak G. N. Schmalz L. Katz S. L. MacLean J. E.. (2018). Longitudinal changes in clinical characteristics and outcomes for children using long-term non-invasive ventilation. PLoS ONE. 13, e0192111. 10.1371/journal.pone.0192111 29381756 PMC5790245

[B13] Colvin K. L. Yeager M. E. What people with Down syndrome can teach us about cardiopulmonary disease. *Eur. Resp. Rev*. (2017) 26, 16. 10.1183/16000617.0098-2016 28223397 PMC9489111

[B14] da Rocha M. Ferraz R. C. M. Guo Chen V. Antonio Moreira G. Raimundo Fujita R. (2017). Clinical variables determining the success of adenotonsillectomy in children with Down syndrome. Int. J. Pediatr. Otorhinolaryngol. 102, 148–153. 10.1016/j.ijporl.2017.09.017 29106863

[B15] Danopoulos S. Deutsch G. H. Dumortier C. Mariani T. J. Al Alam D. (2021). Lung disease manifestations in Down syndrome. Am. J. Physiol. Lung Cell Mol. Physiol. 321, L892–L899. 10.1152/ajplung.00434.2020 34469245 PMC8616621

[B16] Dimopoulos K. Constantine A. Clift P. Condliffe R. Moledina S. Jansen K.. (2023). Cardiovascular complications of Down syndrome: Scoping review and expert consensus. Circulation 147, 425–441. 10.1161/CIRCULATIONAHA.122.059706 36716257 PMC9977420

[B17] Dudoignon B. Amaddeo A. Frapin A. Thierry B. de Sanctis L. Arroyo J. O.. (2017). Obstructive sleep apnea in Down syndrome: benefits of surgery and noninvasive respiratory support. Am. J. Med. Genet. A 24, 24. 10.1002/ajmg.a.38283 28544488

[B18] Focus on Geography Series (2022). 2021 census of population: Alberta, province. Available online at: https://www12.statcan.gc.ca/census-recensement/2021/as-sa/fogs-spg/page.cfm?r=1andLang=Eanddguid=2021A000248andTOPIC=1 (accessed March 24, 2023).

[B19] Harris P. A. Taylor R. Thielke R. Payne J. Gonzalez N. Conde J. G.. (2009). Research electronic data capture (redcap)—A metadata-driven methodology and workflow process for providing translational research informatics support. J. Biomed. Inform. 42, 377–381. 10.1016/j.jbi.2008.08.010 18929686 PMC2700030

[B20] Hill C. M. Evans H. J. Elphick H. Farquhar M. Pickering R. M. Kingshott R. Prevalence predictors of obstructive sleep apnoea in young children with Down syndrome. Sleep Med. (2016) 27, 99–106. 10.1016/j.sleep.2016.10.001. 10.1016/j.sleep.2016.10.001 27938928

[B21] Hudson S. Abusido T. Sebastianski M. Castro-Codesal M. L. Lewis M. MacLean J. E.. (2022). Long-term non-invasive ventilation in children with Down syndrome: a systematic review. Front. Pediat. 10, 886727. 10.3389/fped.2022.886727 35676906 PMC9168004

[B22] Iber C. Ancoli-Israel S. Chesson A. Quan S. (2007). The AASM Manual for the Scoring of Ssleep and Associated Events American Academy of Sleep Medicine. Westchester: American Academy of Sleep Medicine.

[B23] Jayaratne Y. S. N. Elsharkawi I. Macklin E. A. Voelz L. Weintraub G. Rosen D.. (2017). The facial morphology in Down syndrome: a 3D comparison of patients with and without obstructive sleep apnea. Am. J. Med. Genet. A 173, 3013–3021. 10.1002/ajmg.a.38399 28815893 PMC5650534

[B24] Katz S. L. Kirk V. G. MacLean J. E. Bendiak G. N. Harrison M. A. Barrowman N.. (2020). Factors related to positive airway pressure therapy adherence in children with obesity and sleep-disordered breathing. J. Clin. Sleep Med. 16, 733–741. 10.5664/jcsm.8336 32029068 PMC7849790

[B25] Konstantinopoulou S. Tapia I. E. Kim J. Y. Xanthopoulos M. S. Radcliffe J. Cohen M. S.. (2016). Relationship between obstructive sleep apnea cardiac complications and sleepiness in children with Down syndrome. Sleep Med. 17, 18–24. 10.1016/j.sleep.2015.09.014 26847969

[B26] Lal C. White D. R. Joseph J. E. van Bakergem K. LaRosa A. (2015). Sleep-disordered breathing in Down syndrome. Chest 147, 570–579. 10.1378/chest.14-0266 25644910

[B27] Lee C. F. Lee C. H. Hsueh W. Y. Lin M. T. Kang K. T. (2018). Prevalence of obstructive sleep apnea in children with Down syndrome: a meta-analysis. J. Clin Sleep Med. 14, 867–875. 10.5664/jcsm.7126 29734982 PMC5940439

[B28] MacDonagh L. Farrell L. O'Reilly R. McNally P. Javadpour S. Cox D. W.. (2021). Efficacy and adherence of noninvasive ventilation treatment in children with Down syndrome. Pediatr. Pulmonol. 56, 1704–1715. 10.1002/ppul.25308 33730448

[B29] MacLean J. E. Fauroux B. Long-term non-invasive ventilation in children: transition from hospital to home. Paediat. Respirat. Rev. (2023). 10.1016/j.prrv.2023.01.002 36806331

[B30] Marcus C. L. Brooks L. J. Draper K. A. Gozal D. Halbower A. C. Jones J.. (2012). Diagnosis and management of childhood obstructive sleep apnea syndrome. Pediatrics 130, 576–584. 10.1542/peds.2012-1671 22926173

[B31] Marcus C. L. Omlin K. J. Basinki D. J. Bailey S. L. Rachal A. B. Von Pechmann W. S.. (1992). Normal polysomnographic values for children and adolescents. Am. Rev. Respir. Dis. 146, 1235–1239. 10.1164/ajrccm/146.5_Pt_1.1235 1443877

[B32] Maris M. Verhulst S. Wojciechowski M. Van de Heyning P. Boudewyns A. (2016). Prevalence of obstructive sleep apnea in children with Down syndrome. Sleep 39, 699–704. 10.5665/sleep.5554 26612391 PMC4763351

[B33] Maris M. Verhulst S. Wojciechowski M. Van de Heyning P. Boudewyns A. (2017). Outcome of adenotonsillectomy in children with Down syndrome and obstructive sleep apnoea. Arch. Dis. Child. 102, 331–336. 10.1136/archdischild-2015-310351 27484971

[B34] Morrison L. Suresh S. Leclerc M. J. Kapur N. (2022). Symptom care approach to noninvasive ventilatory support in children with complex neural disability. J. Clin. Sleep Med. 18, 1145–1151. 10.5664/jcsm.9836 34928205 PMC8974367

[B35] Nathan A. M. Loo H. Y. de Bruyne J. A. Eg K. P. Kee S. Y. Thavagnanam S.. (2017). Thirteen years of invasive and noninvasive home ventilation for children in a developing country: a retrospective study. Pediat. Pulmonol. 52, 500–507. 10.1002/ppul.23569 27712049

[B36] Nerfeldt P. Sundelin A. (2020). Obstructive sleep apnea in children with Down syndrome—Prevalence and evaluation of surgical treatment. Int. J. Pediatr. Otorhinolaryngol. 133, 109968. 10.1016/j.ijporl.2020.109968 32126418

[B37] O'Donnell A. R. Bjornson C. L. Bohn S. G. Kirk V. G. (2006). Compliance rates in children using noninvasive continuous positive airway pressure. Sleep 29, 651–658. 10.1093/sleep/29.5.651 16774155

[B38] Ong A. A. Atwood C. M. Nguyen S. A. Teufel R. J. Lal C. LaRosa A. C.. (2018). Down syndrome and pediatric obstructive sleep apnea surgery: a national cohort. Laryngoscope 128, 1963–1969. 10.1002/lary.27063 29280489

[B39] Pascoe J. E. Sawnani H. Hater B. Sketch M. Modi A. C. (2019). Understanding adherence to non-invasive ventilation in youth with duchenne muscular dystrophy. Pediatr. Pulmonol. 54, 2035–2043. 10.1002/ppul.24484 31475475 PMC6851431

[B40] Perriol M. P. Jullian-Desayes I. Joyeux-Faure M. Bailly S. Andrieux A. Ellaffi M.. (2019). Long-term adherence to ambulatory initiated continuous positive airway pressure in non-syndromic osa children. Sleep Breath. 23, 575–578. 10.1007/s11325-018-01775-2 30685850

[B41] Rabes L. Adan-Lirola L. Gonzalez-Molina M. D. P. Galvan-Roman J. M. Moldenhauer F. Roy-Vallejo E.. (2022). Evaluation of congenital and acquired heart diseases in a spanish cohort of adults with Down syndrome. Sci. Rep. 12, 22461. 10.1038/s41598-022-26918-0 36577781 PMC9795113

[B42] Rechtschaffen A. K. (1968). A Manual of Standardized Terminology, Techniques and Scoring System for Sleep Stages of Human Subjects. University of California, Los Angeles: U. S. National Institute of Neurological Diseases and Blindness, Neurological Information Network.11422885

[B43] Shete M. M. Stocks R. M. Sebelik M. E. Schoumacher R. A. (2010). Effects of adeno-tonsillectomy on polysomnography patterns in Down syndrome children with obstructive sleep apnea: a comparative study with children without Down syndrome. Int. J. Pediatr. Otorhinolaryngol. 74, 241–244. 10.1016/j.ijporl.2009.11.006 20097432

[B44] Tadic M. Gherbesi E. Faggiano A. Sala C. Carugo S. Cuspidi C.. (2022). The impact of continuous positive airway pressure on cardiac mechanics: Findings from a meta-analysis of echocardiographic studies. J. Clin. Hyperten. 24, 795–803. 10.1111/jch.14488 35695237 PMC9278581

[B45] Takizawa H. Takahashi M. Maki K. (2022). Three-dimensional assessment of craniofacial features in patients with Down syndrome during the mixed dentition period: a case-control study. Cleft. Palate. Craniofac. J. 59, 177–184. 10.1177/1055665621998181 33685243

[B46] Tanaka O. Amano Y. Uchida A. Moriya K. Takahashi K. Nara N.. (2020). Changes in right-to-left cardiac shunting by continuous positive airway pressure: a word of caution. J. Neurol. Sci. 413, 116765. 10.1016/j.jns.2020.116765 32160571

[B47] Waters K. A. Castro C. Chawla J. (2020). The spectrum of obstructive sleep apnea in infants and children with Down syndrome. Int. J. Pediatr. Otorhinolaryngol. 129, 109763. 10.1016/j.ijporl.2019.109763 31704574

